# Supplementary material for: Clinical Significance of Tumor Size in Gross Extrathyroidal Extension to Strap Muscles (T3b) in Papillary Thyroid Carcinoma: Comparison with T2
Source: Cancers (Basel). 2022 Sep 23;14(19):4615. doi: 10.3390/cancers14194615 (PMC9563603; doi:10.3390/cancers14194615)
Supplement: Supplementary file 1 [file cancers-14-04615-s001.zip › cancers-1920620-supplementary.pdf]

[Original tables with all information including non-significant variables]

Table S1. Univariate and multivariate analyses of recurrence risk factors in patients with T2 and T3b including non-significant variables.

	Univariate		Multivariate	
	**HR (95% CI)**	**p-value**	**HR (95% CI)**	**p-value**
**Age**	0.958(0.934-0.982)	0.001	0.968(0.944-0.993)	0.012
**Gender**				
Female	ref.			
Male	2.830 (1.445-5.544)	0.002	2.506(1.277-4.918)	0.008
**Extent of surgery**				
Lobectomy	ref.			
TT and/or mRND	1.016(0.495-2.084)	0.966		
**Tumor size**	1.656 (1.206-2.275)	0.002	1.434(1.036-1.984)	0.030
**Multifocality**	1.081(0.551-2.119)	0.821		
**Bilaterality**	1.314(0.650-2.655)	0.447		
**Lymphatic invasion**	6.129(2.372-15.833)	<0.001	4.822(1.851-12.562)	0.001
**Vascular invasion**	0.737(0.177-3.076)	0.676		
**$BRAF^{V600E}$ positivity**	0.674(0.282-1.614)	0.376		
**T stage**				
T2	ref.			
T3b	1.185 (0.604-2.324)	0.621		
**N stage**		<0.001		
N0	ref.		Ref.	0.175
N1a	8.219 (1.927-35.053)	0.004	3.588(0.728-17.672)	0.116
N1b	8.592 (1.904-39.764)	0.005	2.260(0.416-12.275)	0.345

Data are expressed as hazard ratio (HR) and 95% confidence interval (CI). A statistically significant difference was defined as $p < 0.05$. Abbreviations: TT, total thyroidectomy; mRND, modified radical neck dissection; T, tumor; N, node.

Table S2. Univariate and multivariate analyses of recurrence risk factors in patients with T2 and T3b-1 including non-significant variables.

	Univariate		Multivariate	
	**HR (95% CI)**	**p-value**	**HR (95% CI)**	**p-value**
**Age**	0.938(0.907-0.969)	<0.001	0.955(0.923-0.988)	0.008
**Gender**				
Female	ref.			
Male	2.290 (0.989-5.301)	0.053		
**Extent of surgery**				
Lobectomy	ref.			
TT and/or mRND	0.606(0.263-1.397)	0.240		
**Tumor size**	1.626(1.000-2.646)	0.050	1.574(0.923-2.682)	0.096
**Multifocality**	0.476(0.186-1.217)	0.121		
**Bilaterality**	0.428(0.127-1.446)	0.172		
**Lymphatic invasion**	5.599(1.895-16.544)	0.002	2.653(0.839-8.385)	0.097
**Vascular invasion**	0.611(0.082-4.539)	0.630		
**$BRAF^{V600E}$ positivity**	0.876(0.282-2.716)	0.818		
**T stage**				
T2	ref.			
T3b	0.556 (0.218-1.422)	0.221		
**N stage**				
N0	ref.	0.001		0.093
N1a	12.957 (1.718-97.707)	0.013	6.581(0.778-55.638)	0.084
N1b	11.026 (1.288-94.378)	0.028	4.332(0.441-42.599)	0.209

Data are expressed as hazard ratio (HR) and 95% confidence interval (CI). A statistically significant difference was defined as $p < 0.05$. Abbreviations: TT, total thyroidectomy; mRND, modified radical neck dissection; T, tumor; N, node.

Table S3. Univariate and multivariate analyses of recurrence risk factors in patients with T2 and T3b-2 including non-significant variables.

	**Univariate**		**Multivariate**	
	**HR (95% CI)**	**p-value**	**HR (95% CI)**	**p-value**
**Age**	0.957(0.930-0.985)	0.003	0.959(0.932-0.987)	0.004
**Gender**				
Female	ref.			
Male	1.541 (0.708-3.357)	0.276		
**Extent of surgery**				
Lobectomy	ref.			
TT and/or mRND	1.146 (0.527-2.496)	0.731		
**Tumor size**	1.218 (0.592-2.508)	0.593		
**Multifocality**	1.207(0.558-2.609)	0.633		
**Bilaterality**	1.700(0.758-3.814)	0.198		
**Lymphatic invasion**	4.961(1.871-13.158)	0.001	3.208(1.159-8.882)	0.025
**Vascular invasion**	0.497(0.067-3.669)	0.497		
**$BRAF^{V600E}$ positivity**	0.913(0.329-2.535)	0.861		
**T stage**				
T2	ref.		ref	
T3b	2.640 (1.198-5.817)	0.016	2.659(1.159-6.099)	0.021
**N stage**				
N0	ref.	0.001	ref.	0.296
N1a	6.965 (1.593-30.460)	0.010	3.079(0.611-15.523)	0.173
N1b	8.412 (1.817-38.933)	0.006	2.051(0.337-12.488)	0.436

Data are expressed as hazard ratio (HR) and 95% confidence interval (CI). A statistically significant difference was defined as $p < 0.05$. Abbreviations: TT, total thyroidectomy; mRND, modified radical neck dissection; T, tumor; N, node.

Table S4. Comparison of recurrence rate between patients with T1 and T3b-1 disease.

	**T1 (*n* = 5280)**	**T3b-1 (*n* = 217)**	**p-value**
**Recurrence**	130(2.5%)	6(2.8%)	0.778

Data are expressed as hazard ratio (HR) and 95% confidence interval (CI). A statistically significant difference was defined as $p < 0.05$. Abbreviations: T, tumor.

Table S5. Univariate Cox regression analyses of T stage as a risk factor for recurrence in patients with T1 and T3b-1.

	**Univariate**	
	**HR (95% CI)**	**p-value**
**T stage**		
T1	ref.	
T3b-1	1.118(0.493-2.533)	0.790

Data are expressed as hazard ratio (HR) and 95% confidence interval (CI). A statistically significant difference was defined as $p < 0.05$. Abbreviations: T, tumor.
